# Broadband Variable Meta-Axicons Based on Nano-Aperture Arrays in a Metallic Film

**DOI:** 10.1038/s41598-018-29265-1

**Published:** 2018-08-02

**Authors:** Yunzhi Zhu, Dunzhao Wei, Zeyu Kuang, Qianjin Wang, Yongmei Wang, Xiaoyang Huang, Yong Zhang, Min Xiao

**Affiliations:** 1 National Laboratory of Solid State Microstructures, College of Engineering and Applied Sciences, and School of Physics, Nanjing University, Nanjing, 210093 China; 2 Department of Physics, University of Arkansas, Fayetteville, Arkansas 72701 USA

## Abstract

Metasurfaces are two-dimensional metamaterials composed of a carefully designed series of subwavelength meta-atom (antenna or aperture) arrays. These surfaces can manipulate the phase, amplitude and polarization of output light by changing the shapes and orientations of the meta-atoms on a subwavelength scale. Using these properties, we experimentally demonstrate variable meta-axicons composed of rectangular nano-apertures arranged in several concentric rings that can focus left circularly polarized (LCP) light into a real Bessel beam and defocus right circular polarized (RCP) light to form a virtual beam. A desired phase discontinuity in cross-polarized transmitted light is introduced along the interface by controlling the orientations of the nano-apertures. In addition, the meta-axicons can generate Bessel beams of arbitrary orders by suitable design of the phase profile along the surface. The meta-axicons demonstrate broadband optical properties that can switch the wavelength of the incident light from 690 nm to 1050 nm. These variable meta-axicons open a path towards the development of new applications using integrated beam shaping devices.

## Introduction

In 1987, Durnin^1^ first proposed that Bessel beams were non-diffraction mode solutions to the Helmholtz equation with self-constructing characteristics, which meant that their transverse intensity distributions remained invariant during propagation in free space. An ideal Bessel beam is unbounded and therefore requires infinite energy. In reality, we can only produce reasonably well-approximated Bessel beams with little or no diffraction over limited propagation distances. These beams have been explored for use in numerous fields, including nonlinear optics^2,3^, ultrasonic medical diagnosis^4^, optical trapping^5^, telescopes^6^ and optical communications applications^7,8^. In particular, high-order Bessel beams have been attracting increasing interest because they can carry the orbital angular momentum (OAM) of light^9,10^. Two approaches are typically used to generate zero-order Bessel beams: illumination of an annular slit located in the rear focal plane of a lens using a plane wave^11^ or use of an axicon^12^, which is a conically shaped lens, to refract all incident plane waves symmetrically towards the optical axis. A high-order Bessel beam can generally be obtained by illuminating an axicon using a Laguerre-Gauss beam^13^ or by propagating a zero-order Bessel beam through a phase-modulating element such as a spatial light modulator^14^. However, these approaches are dependent on relatively cumbersome devices, which would undoubtedly limit the applications of Bessel beams in miniaturized optical systems.

Recently, metasurfaces have attracted significant research interest because they offer considerable flexibility in the engineering of their electromagnetic properties. The unique optical properties of metasurfaces will enable the realization of many novel phenomena and functionalities that do not normally exist in natural materials^15–17^. Metasurfaces have therefore been widely used in applications including nonlinear photonics^18–21^, optical OAM^18,22–26^, optical rotation^27^, invisibility cloaking^28–30^, metalenses^31,32^ and holography^33,34^. These surfaces are generally composed of a series of subwavelength meta-atom (antenna or aperture) arrays that have carefully designed shapes and orientations. Phase discontinuities are then introduced when light propagates through an interface between two media^35^. The Pancharatnam–Berry phase^36,37^, which is also called the geometric phase, is a widely used method for performing phase modulation via the space-variant optical axis orientations of meta-atoms. Based on these properties, one interesting potential application of metasurfaces is the formation of a meta-axicon^38–40^.

In this paper, we propose a broadband variable meta-axicon that is composed of a set of nano-apertures within a thin metallic film. Based on Babinet’s principle^41^, we use plasmonic rectangular nano-apertures that have been arranged in concentric rings with different orientations to create a variable meta-axicon with focusing characteristics that are dependent on the polarization direction of the incident light. The proposed dual-polarity meta-axicon can produce real or virtual high-order Bessel beams and thus has potential for application to integrated beam shaping devices for use in high-quality imaging of inhomogeneous media^42,43^ and living cells^44^. In addition, the working wavelength of our meta-axicon extends to the near-infrared band, which could be useful in high-resolution multi-photon imaging applications.

## Results

### Zero-order Bessel beam

**Figure 1:**
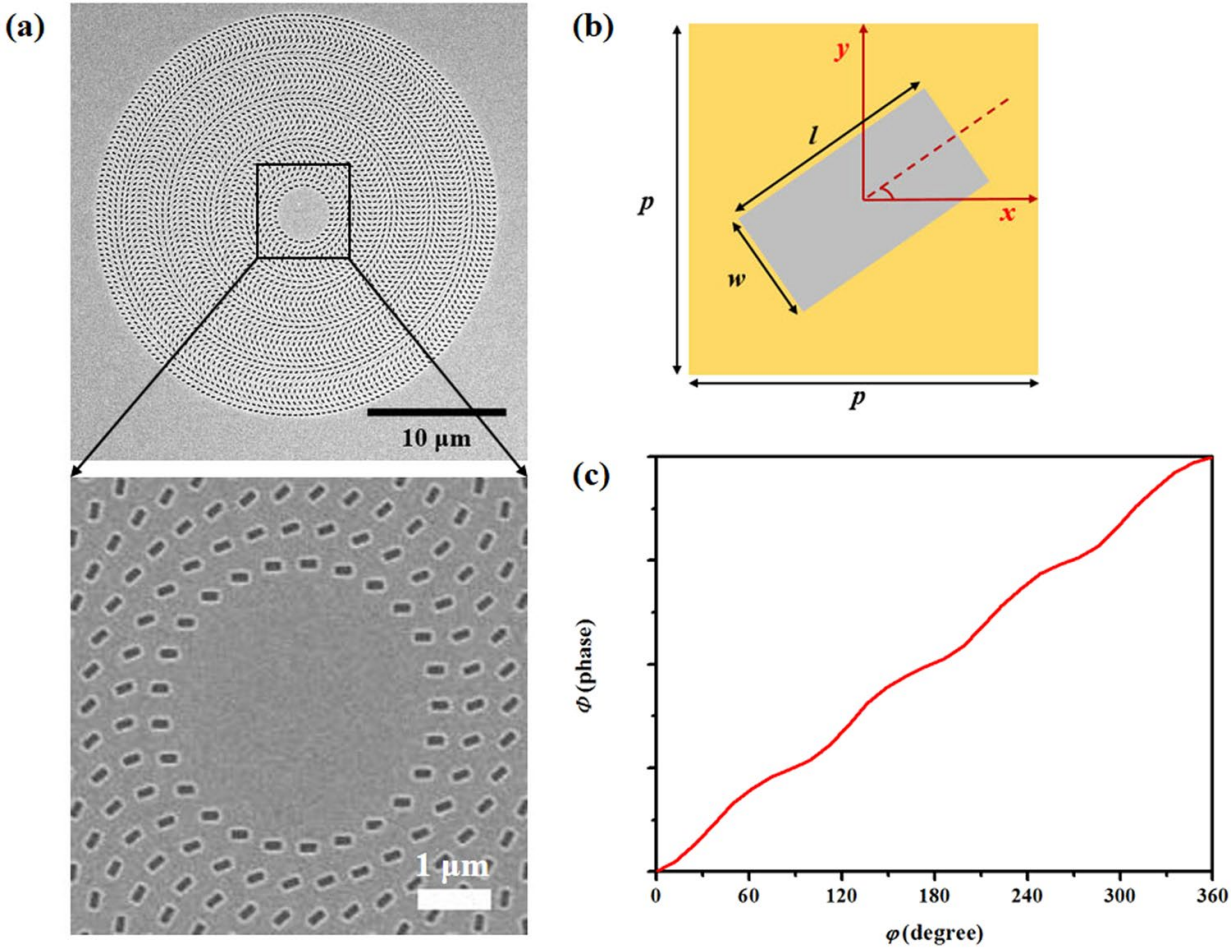
**(a)** SEM images of the proposed structure that was used to generate zero-order Bessel beams. The bottom image shows an enlarged view of part of the meta-axicon that shows the arrangement of the nano-apertures. **(b)** Schematic of a single rectangular nano-aperture. The period *p* is 400 nm. The length and width of the rectangular nano-aperture are *l = *260 nm and *w* = 130 nm, respectively. **(c)** Phase profile *Φ* versus orientation angle *φ* for the rectangular nano-apertures.

First, we demonstrate a meta-axicon for use in the generation of a zero-order Bessel beam. The structure that is used in this design is composed of rectangular nano-apertures arranged in a number of concentric rings and was fabricated in an 80-nm-thick Au film supported by a SiO_2_ substrate. Scanning electron microscope (SEM) images of the nano-aperture array are shown in Fig. 1(a). The ring radius increases with step sizes of 400 nm and the distance between the pairs of neighbouring nano-apertures in each ring is also 400 nm. The rectangular nano-apertures in each ring all have the same orientation angle; however, there is an angular difference *φ*_0_ of 24° between the nano-apertures in neighbouring rings. A single rectangular nano-aperture has dimensions of 260 × 130 nm^2^. The meta-axicon is 28 μm in diameter. In our design, the starting point for the Bessel beam is set to be 7.5 μm away from the sample surface. Therefore, the radius of the unstructured area at the center can be calculated to be *r*_0_ = 2 μm based on the generalized Snell’s law^33^. A schematic diagram of a single nano-aperture is shown in Fig. 1(b). This aperture can be rotated within the *x*-*y* plane to an orientation angle of *φ* to produce a specific phase delay.

**Figure 2:**
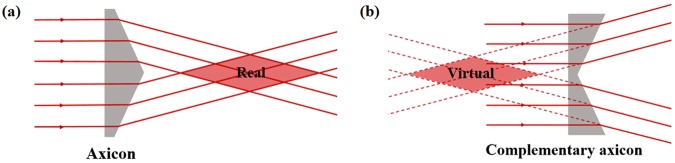
Working principles of **(a)** conventional axicon and **(b)** complementary axicon.

When the nano-aperture is illuminated using left circularly polarized (LCP)/right circularly polarized (RCP) incident light, which is denoted by ${{\boldsymbol{E}}}_{I}^{L/R}$, the transmitted light can be written as^45^:

1 $${{\boldsymbol{E}}}_{T}^{L/R}=\hat{M}\cdot {{\boldsymbol{E}}}_{I}^{L/R}=\frac{{t}_{o}+{t}_{e}}{2}{{\boldsymbol{E}}}_{I}^{L/R}+\frac{{t}_{o}-{t}_{e}}{2}exp(i2m\,\phi ){{\boldsymbol{E}}}_{I}^{L/R},$$

where $\hat{M}$ is a Jones matrix, and ${t}_{o}$ and ${t}_{e}$ are the transmission coefficients of the incident beams, which have linear polarizations oriented along the two principal axes of the metasurface. The transmitted light contains two components: one component is circularly polarized (CP) light, which has the same polarization direction as the incident light, while the other component has the opposite polarization direction and an additional Pancharatnam-Berry phase of *Φ* = 2*mφ*; here, *m* is ‘+1’ for LCP incident light and ‘−1’ for RCP incident light. To verify the above theory, we use the finite-difference time-domain (FDTD) method to simulate the phase shift *Φ* that occurs when the orientation angle *φ* of the nano-apertures is varied. The incident wavelength is set at 800 nm. We first set the LCP light to be the incident light and detected the RCP component in the output port. The simulation results are shown in Fig. 1(c); by varying the orientation angle *φ* of the nano-aperture from 0° to 360°, we can achieve an approximate phase shift of *Φ* within the range from 0 to 4π for RCP transmitted light. The simulated results agree well with the formula *Φ = *2*φ*. Based on the above results, we designed the metasurface geometry (Fig. 1(a)) to realize a Bessel beam. Under irradiation by LCP light, the transmitted RCP light will carry a spatial phase in which the gradient changes along the radius of the metasurface because the orientation angle difference between rectangular apertures in neighbouring rings remains constant. Therefore, under illumination by LCP light, the RCP component of the transmitted light, which is focused using the designed metasurface (i.e., the meta-axicon), forms a real Bessel beam, as shown in Fig. 2(a). In contrast, if we input RCP light and collect the transmitted LCP component, the additional Pancharatnam-Berry phase follows the formula *Φ* = −2*φ*. The metasurface sample acts as a complimentary meta-axicon and defocuses the transmitted LCP component to form another virtual Bessel beam (Fig. 2(b)).

**Figure 3:**
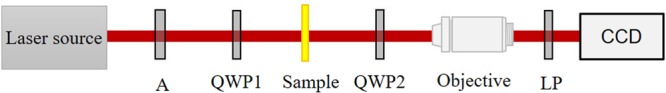
Schematic diagram of the experimental setup. A: attenuator; QWP: achromatic quarter-wave plate; LP: linear polarizer; CCD: charge-coupled device.

To validate the various functions of the meta-axicon in its different configurations, we measured the patterns that were transmitted along the light propagation direction. Figure 3 shows a schematic diagram of the experimental setup. In the experiments, a wavelength of 780 nm is used. The laser light is converted into CP light using the quarter-wave plate (QWP1) that is located in front of the sample. A component with a specific circular polarization is selected using a linear polarizer (LP) and a quarter-wave plate (QWP2), and the imaged patterns are then recorded using a charge-coupled device (CCD) camera.

**Figure 4:**
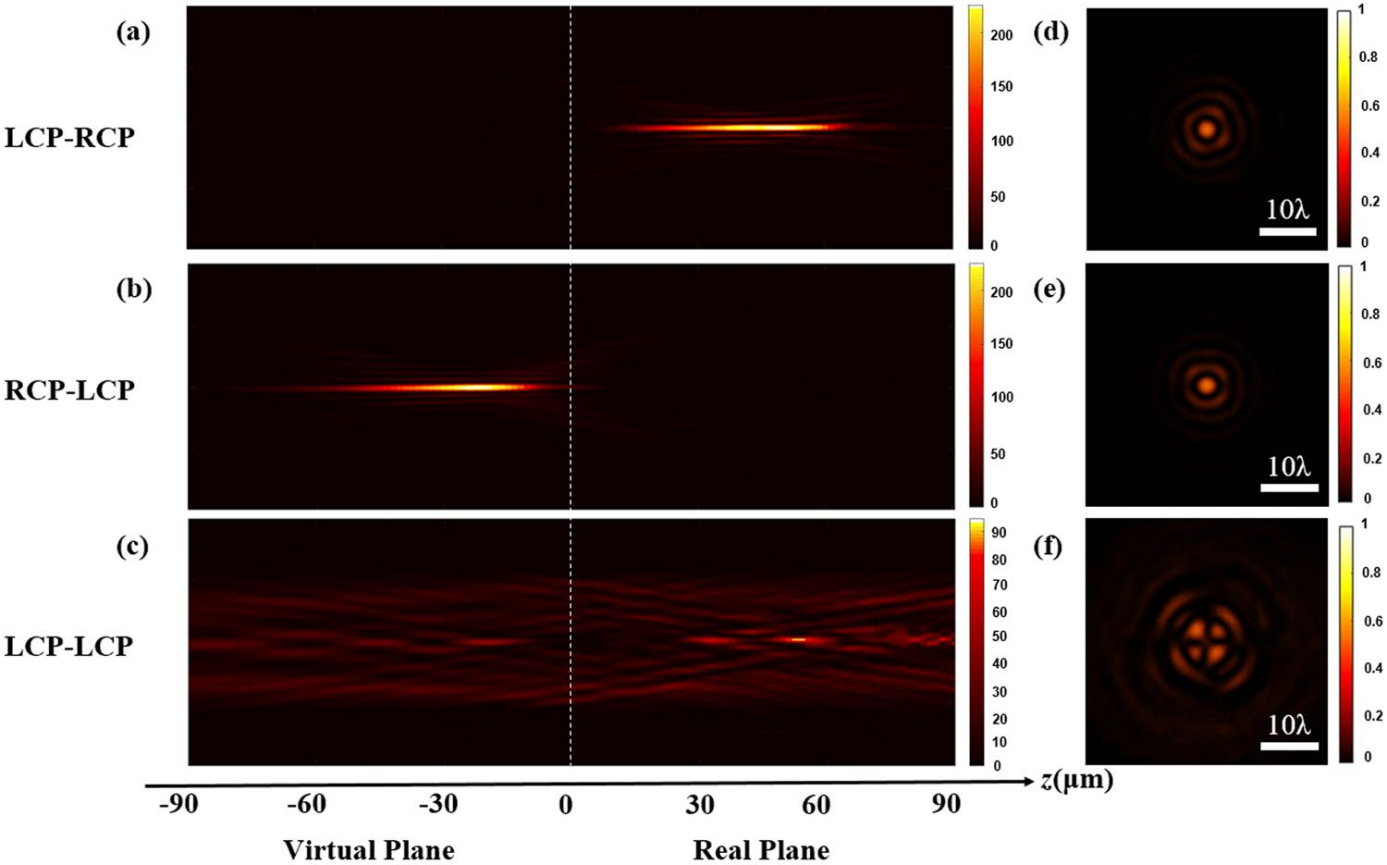
Intensity distributions of the meta-axicon for three different incident/transmitted light combinations at an incident wavelength of 780 nm: **(a)** LCP/RCP, **(b)** RCP/LCP, and **(c)** LCP/LCP. **(d–f)** Cross-sections of the three cases described above, where their positions are *z* = 48 μm, −23 μm and 48 μm, respectively. The dotted line represents the sample plane in each case.

Three different incident/transmitted light combinations were measured, including LCP/RCP, RCP/LCP and LCP/LCP, and the measured results for these combinations are shown in Fig. 4(a,b and c), respectively. In Fig. 4(a), a real Bessel beam is generated on the output side, which indicates that the meta-axicon is positive for the LCP incident beam. However, in Fig. 4(b), when we input the RCP light, the transmitted LCP light is divergent and a virtual image of the Bessel beam is shown in the virtual plane; this indicates that the metasurface has become a complementary meta-axicon for the RCP incident beam. In addition, when we input an LCP beam and detect the same polarization component as that of the input beam at the output port, there is no Bessel beam, as shown in Fig. 4(c). Figure 4(d and e) show the brightest cross-sections of the real and virtual Bessel beams, i.e., at *z* = 48 μm and −23 μm, respectively. The difference between their central positions (see Fig. 4(a and b)) is caused by nonparallel incident light. Typical Bessel beam profiles are obviously shown in Fig. 4(d and e). The cross-section at *z* = 48 μm in Fig. 4(c) is also shown in Fig. 4(f) for comparison, i.e., no Bessel beams are observed when using the LCP/LCP configuration. Clearly, the function of the proposed zero-order meta-axicon is variable and shows a dependence on the light polarization direction.

**Figure 5:**
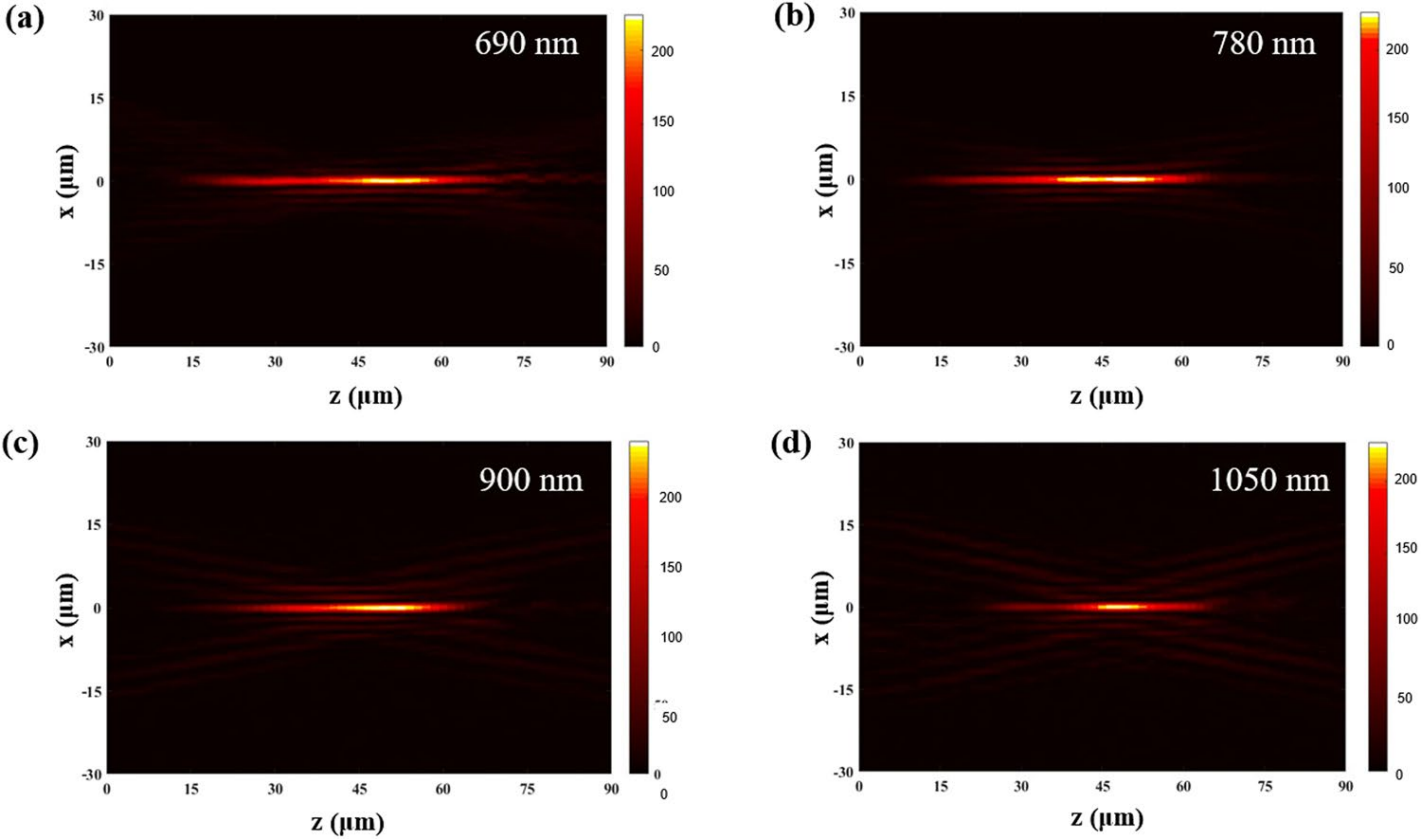
Intensity distributions of RCP light when transmitted through the meta-axicon under LCP incidence conditions at incident wavelengths of **(a)** 690 nm, **(b)** 780 nm, **(c)** 900 nm and **(d)** 1050 nm.

Our meta-axicon also demonstrates broadband characteristics and can maintain high-level beam quality over the wavelength range from 690 nm to 1050 nm. We measured the broadband characteristics of the meta-axicon, which can generate zero-order Bessel beams using LCP light at different incident wavelengths, and the results are as shown in Fig. 5. The depth of focus in each of these four images decreases with increasing wavelength. This occurs because the maximum no-diffraction length *z*_max_ for a Bessel beam is given by^46^:

2 $${z}_{{\rm{\max }}}=\frac{Rd}{\lambda },$$

where *λ* is the wavelength, *R* is the radial width of the meta-axicon and *d* is the radial period when the changed phase covers the 2*π* range; in our experiments, *R = *12 μm, and *d* = 3 μm. Therefore, the z_max_ values from the theory are 52 μm, 46 μm, 40 μm and 34 μm, and those from our experimental results are 49 μm, 46.5 μm, 38 μm and 33 μm for incident wavelengths of 690 nm, 780 nm, 900 nm and 1050 nm, respectively. The experimental results thus agree well with the theoretical predictions within the allowable error range. In addition, the central positions of the Bessel beams in the four cases above are all at approximately *z* = 45 μm. This can be attributed to the fact that the additional phase in the transmitted light is related to the orientation angle *φ* of the nano-aperture rather than to the wavelength^47^.

### Higher-order Bessel beams

**Figure 6:**
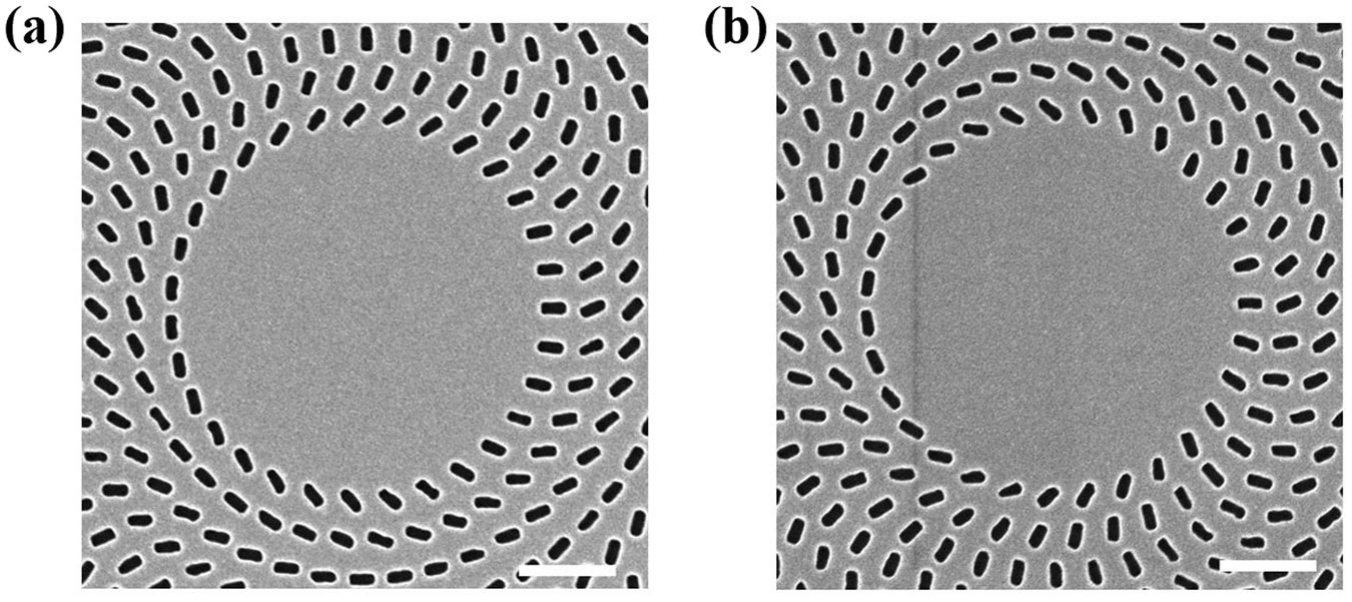
SEM images of higher-order meta-axicons with topological charges of **(a)** *n* = 1 and **(b)** *n* = 3. The scale bar represents 1 μm.

In the design of the meta-axicon for higher-order Bessel beams, we need to combine two types of phase profiles^13,14^. One is the radial phase profile *Φ*_1_, which is used to generate the zero-order Bessel beam, and the other is the azimuthal phase profile *Φ*_2_, which can introduce a vortex phase into the transmitted light through optical spin-orbital angular momentum conversion. The topological charge of the generated high-order Bessel beam can be designed specifically in the profile *Φ*_2_. The overall phase distribution *Φ* of the nano-apertures in the meta-axicon can be expressed as

3 $${\Phi }({\rm{r}})={{\Phi }}_{1}({\rm{r}})+{{\Phi }}_{2}({\rm{r}})$$

where *Φ*_1_(r) = 2*φ*_0_ · $(\frac{\sqrt{{x}^{2}+{y}^{2}}-{r}_{0}}{p})$ and *Φ*_2_(r) = *n* arctan $(\frac{y}{x})$. Here, *φ*_0_ is the angular difference between the nano-apertures in the neighbouring rings of the zero-order meta-axicon; ${r}_{0}$ is the radius of the unstructured area at the center, *p* is the distance between the nano-apertures in neighbouring rings, and *n* is the topological charge that represents the order of the Bessel beam. SEM images of two high-order meta-axicons (with *n* = 1 and *n* = 3) are shown in Fig. 6.

**Figure 7:**
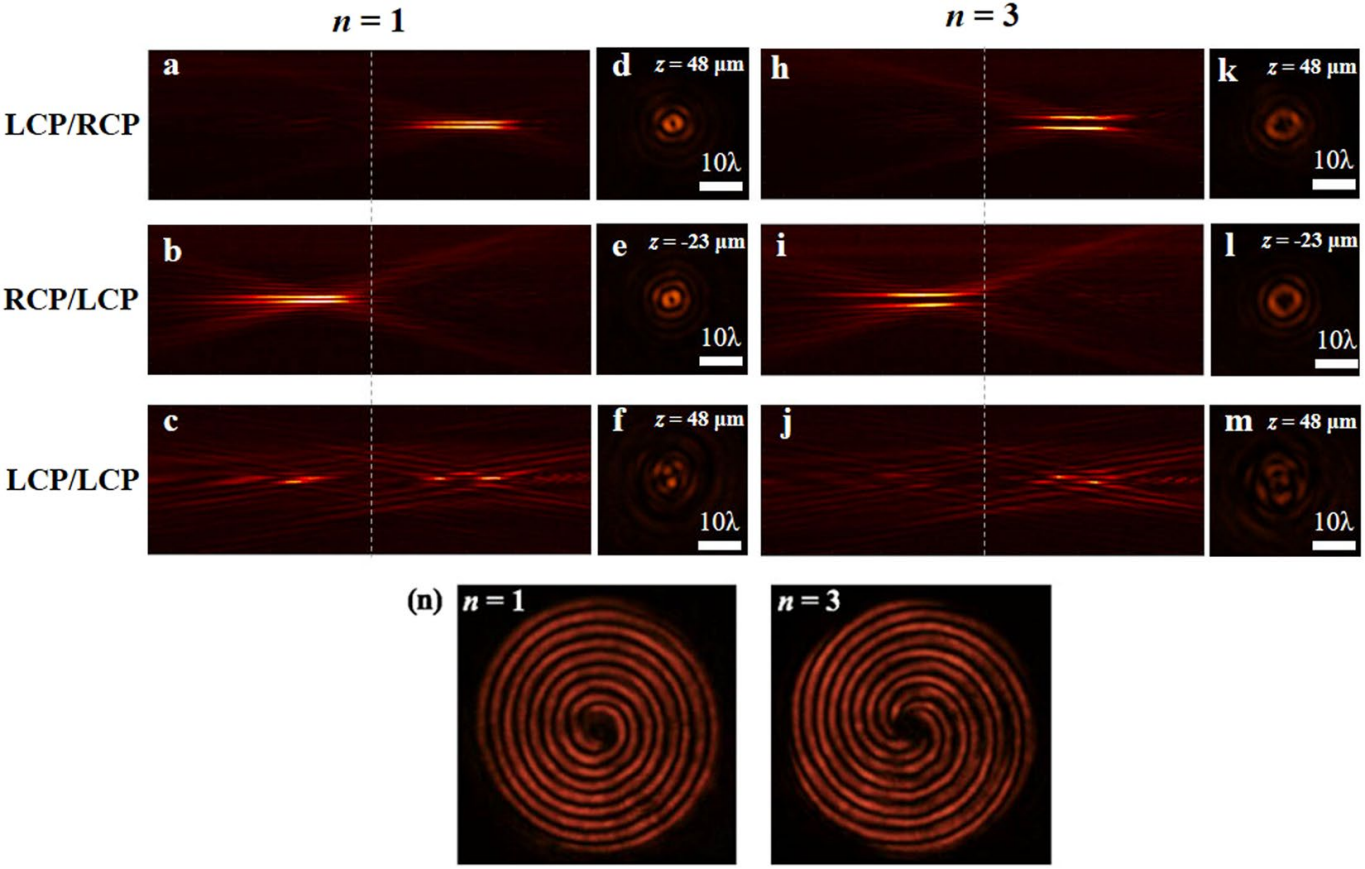
(**a**–**c**) and **(h**–**j)** Intensity profiles of higher-order Bessel beams along the propagation direction with different numbers of topological charges *n*, where the wavelength *λ *= 780 nm. **(d**–**f)** and **(k**–**m)** Cross-sections of (**a**–**c**) and (**h**–**j**), respectively. The dotted lines represent the sample plane. **(n)** Interference patterns of higher-order Bessel beams with topological charges of *n* = 1 (left) and *n* = 3 (right).

In the experiments, we investigate such high-order meta-axicons using an input wavelength of 780 nm. The experimental setup is similar to that for the zero-order meta-axicon (Fig. 3). Different incident/transmitted light combinations (LCP/RCP, RCP/LCP, and LCP/LCP) are again tested. The propagation patterns of the high-order meta-axicons with topological charges of *n* = 1 and *n* = 3 are shown in Fig. 7(a–c and h–j), respectively. The corresponding cross-sections are shown in Fig. 7(d–f and k–m), respectively. Clearly, real and virtual Bessel beams are produced by the LCP/RCP and RCP/LCP configurations, respectively. No specific beam is generated by the LCP/LCP combination. The variable function is also applicable for high-order meta-axicons.

## Discussion

**Figure 8:**
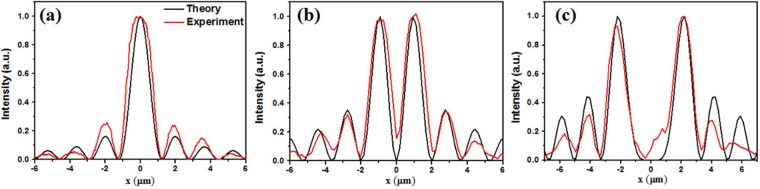
Normalized intensity profiles of the (**a**) zeroth-, (**b**) first- and (**c**) third-order Bessel beams from theory (black) and from experiments (red).

Normally, a higher-order Bessel beam is characterized by a hollow centre, which is a typical characteristic of OAM-carrying beams, as depicted in Fig. 7(d–e and k–l). The beam size, which is defined as the radius of the innermost ring, increases with increasing topological charge *n*. In the experiments, the topological charges of the Bessel beam are detected using the interference method. The QWPs in the experimental setup shown in Fig. 3 are removed. We use *y*-polarized light at 780 nm to irradiate the meta-axicons and record the *x*-polarized transmitted light using a CCD camera at the sample plane (*z* = 0). The *y*-polarized input light can be decomposed into its LCP and RCP components, which, after they pass through the meta-axicon, are converted to into RCP and LCP modes carrying OAMs of +*n* and −*n*, respectively. The superposition of these two transmitted modes produces an *x*-polarized light, which presents the spiral pattern shown in Fig. 7(n). The number of spirals in the intensity patterns is double the number of topological charges |*n*|^48^. In addition, we compare the intensity profiles of the experimental Bessel beams with those of the theoretical beams (Fig. 8). The experimental data for the zeroth-, first- and third-order Bessel beams are derived from Figs 4(d), 7d and k), respectively, and they agree well with the theoretically predicted values.

In conclusion, we have experimentally demonstrated variable broadband meta-axicons that can convert Gaussian beams into real or virtual Bessel beams with designed orders by varying the incident light polarization and detecting the cross-polarized components in the transmitted light. The meta-axicons are composed of concentrically-arranged plasmonic nano-apertures. Changes in the orientation of these nano-apertures provide phase shifts that range from 0 to 2π for the transmitted light. These meta-axicons work well over a wide wavelength range from 690 nm to 1050 nm. Unlike conventional glass axicons, these meta-axicons can be used to fabricate on-chip or fiber-embedded optical integrated devices, including nanophotonic couplers and ultra-thin objective lenses.

## Methods

### Sample fabrication

All samples were fabricated on 1-mm-thick double-side polished SiO_2_ substrates. An 80-nm-thick Au film was deposited on a clean substrate by magnetron sputtering in the same sputtering chamber. The rectangular nano-apertures were then milled on the Au film using a focused ion beam system (Strata FIB 201, FEI Company).

### Measurement

The laser source is a tunable Ti: sapphire laser that has an operating wavelength range from 690 nm to 1050 nm. The laser light is converted into CP light using the quarter-wave plate (QWP1) that is located in front of the sample. The transmitted light is collected using an objective (100×/0.7) and a component of the light with a specific circular polarization is selected using a linear polarizer (LP) and another quarter-wave plate (QWP2); the imaged patterns are then recorded using the CCD camera. By changing the imaging plane using increments of 1.5 μm, we can obtain intensity distributions at various distances from the meta-axicon surface on the transmission side.

### Data availability

The data that support the plots within this paper and other findings of this study are available from the corresponding authors upon reasonable request.
